# Associations of low-intensity light physical activity with physical performance in community-dwelling elderly Japanese: A cross-sectional study

**DOI:** 10.1371/journal.pone.0178654

**Published:** 2017-06-09

**Authors:** Kazuhiro P. Izawa, Ai Shibata, Kaori Ishii, Rina Miyawaki, Koichiro Oka

**Affiliations:** 1 Graduate School of Health Sciences, Kobe University, Suma, Kobe, Japan; 2 Faculty of Health and Sport Sciences, University of Tsukuba, Tsukuba, Ibaraki, Japan; 3 Faculty of Sport Sciences, Waseda University, Tokorozawa, Saitama, Japan; University Of São Paulo, BRAZIL

## Abstract

**Background:**

Physical activity and physical performance relate to quality of life, mortality, and morbidity in elderly people. However, little is known about differences in physical performance related to low-intensity light physical activity (LLPA), high-intensity light physical activity (HLPA), and moderate-intensity physical activity (MPA) and how they are separated by sex in elderly populations.

**Aims:**

This study aimed to determine differences in LLPA, HLPA, MPA, and physical performance, and associations between these measures in community-dwelling elderly men and women.

**Methods:**

Physical activity and physical performance such as timed-up-and-go test, one-leg standing time, and maximum gait speed were measured in 181 community-dwelling elderly men (mean age, 75.1 ± 5.3 years) and 109 women (mean age, 73.4 ± 4.8 years) in 2013. Physical activity was classified as LLPA (1.6~1.9 METs of physical activity), HLPA (2.0~2.9 METs of physical activity), and MPA (over 3 METs of physical activity). The association between the values of these three intensities of physical activity in the participants was assessed by Pearson’s correlation coefficients. Multiple linear regression analyses were used to assess the association of physical performance values with the three groups defined by accelerometer-measured physical activity intensity adjusted for sociographic, behavioral, and multiple diseases in the participants.

**Results:**

MPA was beneficially associated with all physical performance indicators in the men (all *P*<0.05) and women (all *P*<0.05). Only HLPA showed significant associations with the timed-up-and-go test (*P* = 0.001) and maximum gait speed (*P* = 0.006) in women.

**Discussion:**

These results may support the notion that not only HLPA in women but MPA in both sexes appears to improve physical performance in elderly populations.

**Conclusion:**

The present study findings provide novel epidemiological evidence for the potential benefits of HLPA in women and also reinforce the potential benefits of MPA in both sexes, which is the mainstay of public health recommendations.

## Introduction

As in several countries, the elderly population in Japan has been increasing owing to aging of the general population [1–4]. This is a growing public health problem as age-related decline in physical performance is related to life motivation, quality of life, and nutrition in the elderly [5–10].

Traditionally, with regard to the intensity of physical activity and health, it has been recommended that adults focus on moderate- to vigorous-intensity activity [11]. However, recent studies have suggested that there has been a shift from focusing on these intensities to the potential of light-intensity physical activity [12,13]. Howard et al. [14] suggested from the cross-sectional findings of their study that low-intensity light physical activity (LLPA), high-intensity light physical activity (HLPA), and moderate-intensity physical activity (MPA) were beneficially associated with cardiometabolic biomarkers such as waist circumstance, C-reactive protein levels, triglycerides, systolic blood pressure, body mass index (BMI), high density lipoprotein cholesterol, and fasting blood glucose. However, it is yet to be elucidated whether light-intensity physical activity is associated with physical performance in elderly populations.

There are sex-related differences in physical activity and physical performance not only for several diseases but also in community-dwelling individuals [14–17]. Therefore, it is important to understand the sex-related differences associated with physical activity and/or daily physical performance when educating or prescribing exercise for the elderly.

Therefore, this study aimed to determine the differences in LLPA, HLPA, MPA, and physical performance between elderly Japanese men and women in a community-dwelling population. In addition, we investigated the relationship between these measures to test our hypothesis that a positive association would exist between LLPA, HLPA, and MPA and physical performance in this study population.

## Methods

### Participants

**Fig 1:**
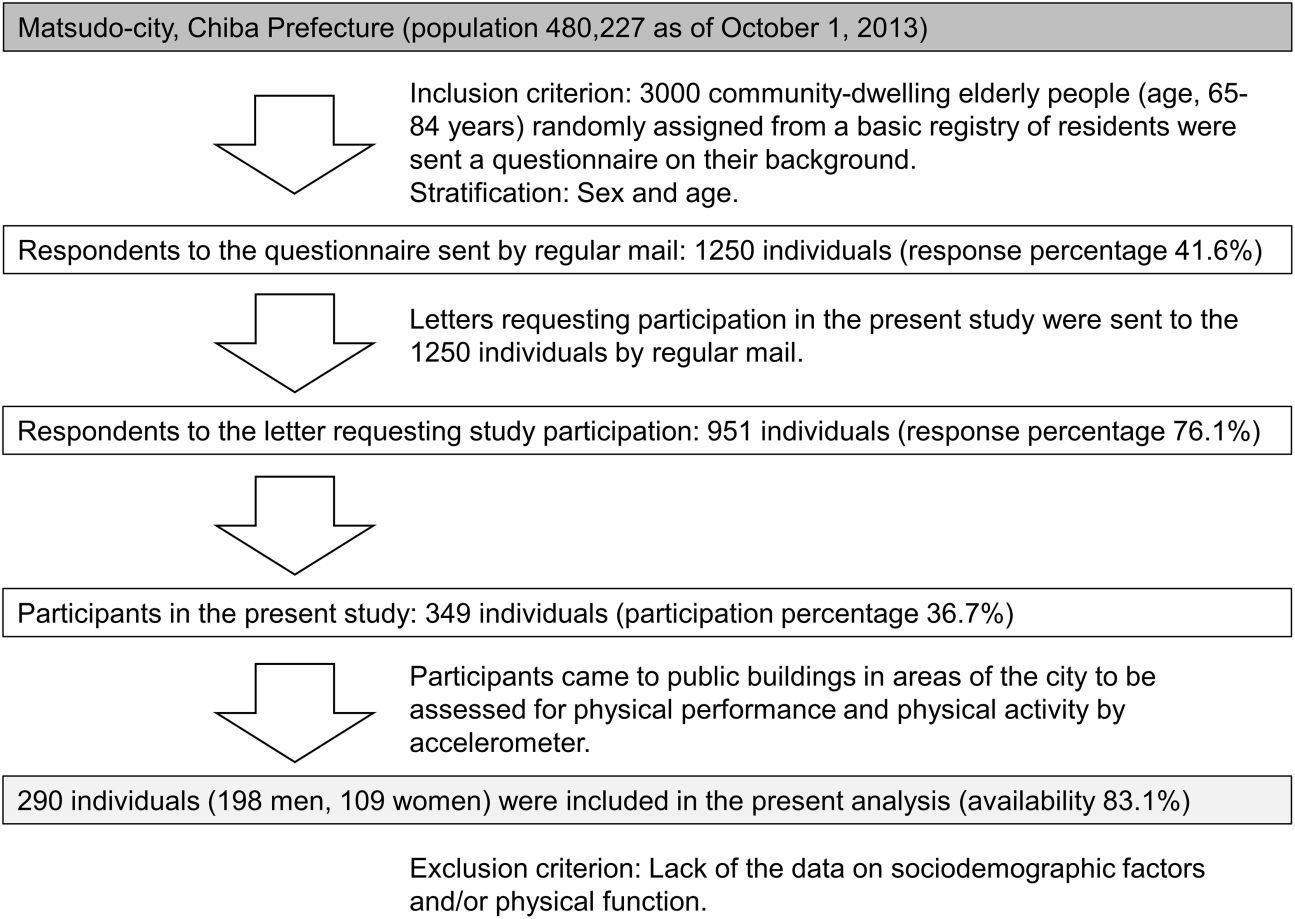
Schematic diagram of the participant selection process.

The sample for this cross-sectional study was selected in Matsudo-city, Chiba Prefecture (population of 480,227 as of October 1, 2013). The inclusion criterion was 3000 community-dwelling elderly people aged 65–84 years who were randomly assigned from a basic registry of residents. They were sent a questionnaire on their background that included questions on age, level of education, marital status, family income, and behavioral factors. In total, 1250 individuals responded to this questionnaire by regular mail (response percentage 41.6%). A letter requesting participation in the present study was then sent to the 1250 responders by regular mail. In total, 951 individuals responded (response percentage 76.1%), but 602 responders declined to participate; thus, 349 individuals were subsequently enrolled in the present study (participation percentage 36.7%). Participants’ physical activity and physical performance were assessed in public buildings. Of the 349 potential participants, 290 were included and 59 were excluded because the data necessary to evaluate their sociodemographic factors, physical activity, and/or physical performance were either incomplete or unavailable. Therefore, 290 participants were included in the present analysis on the basis of the inclusion criteria and available data (Fig 1).

Sociodemographic factors such as age, sex, BMI, level of education (≤12 years), marital status, family income; behavioral factors such as smoking and alcohol intake; and multiple diseases such as hypertension, diabetes, cardiac disease, cerebrovascular disease, gout, osteoporosis, arteriosclerosis obliterans, cancer, and dyslipidemia were identified in the present study. This study was approved by the Waseda University Institutional Committee on Human Research (2013–265). Informed consent was obtained from each participant.

### Physical activity

Physical activity was determined with the HJA-750 C Active style Pro accelerometer (OMRON HEALTHCARE Co., Ltd., Kyoto, Japan), which collected data during waking hours in 60-second epochs for 7 days and was previously validated [18]. Methods of data collection and analysis were performed as previously described [19].

Non-wearing time was defined as intervals of at least 60 consecutive minutes of 0.9 metabolic equivalents (METs) or less based on a validation study [18], with allowance for up to 2 min of observations of some limited movement (≤1.0 METs) within these periods. Days with at least 10 h of wearing time were considered valid. Participants with at least four valid days (10+ h of wearing), including at least 1 weekend day, were included in the analyses. Based on previous studies, physical activity was classified as LLPA (1.6~1.9 METs of physical activity), HLPA (2.0~2.9 METs of physical activity), or MPA (over 3 METs of physical activity) [18, 20, 21].

### Physical performance

Index values of physical performance were determined as those of the timed-up-and-go (TUG) test, one-leg standing time (OLST), and maximum gait speed (MGS). All values were collected by trained investigators based on standard procedures. The TUG test of static and dynamic balance uses the time that a person takes to rise from a chair, walk 3 m, turn around, walk back to the chair, and sit down. The TUG test is used frequently in the elderly population [22]. The time (sec) to complete the TUG was recorded based on the average of two separate trials [22].

OLST was measured by timing the participants from the moment when one leg was raised until it was set back down on the floor, up to a maximum of 60 sec [23–25]. The highest value measurement from two trials was determined. To measure MGS, a 5-m gait test was performed on flat ground along a 5-m line at a gym. Each participant was asked to walk on the 5-m line as fast as possible [26]. An investigator measured the time required for patients to walk from the beginning to the end of the line using a stopwatch. Two trials were performed for MGS. Subsequently, gait speed was calculated as 5 m/time required in sec, and the highest value measured of the two trials was considered to be the gait speed in m/sec [26].

### Statistical analysis

Results are expressed as mean ± standard deviation. Questionnaire data, including sociodemographic factors, behavioral factors, and the total number of multiple diseases, are presented in Table 1. The association between the three values of physical activity intensity in the male and female participants was assessed by Pearson’s correlation coefficients. Separate multiple linear regression models were used to examine the associations of these physical activity values with physical performance values for each sex. Models were adjusted for age, BMI, level of education, marital status, family income ≥¥5,000,000/year, smoking, alcohol intake, and the total number of multiple disease covariates retained in backward elimination in both sexes; *P*<0.2 for retention). A *P* value of <0.05 was considered to indicate statistical significance. Statistical analyses were performed with IBM SPSS 22.0 statistical software (IBM SPSS Japan, Inc., Tokyo, Japan).

**Table 1:** Characteristics of the study population.

Variable	Males	Females
No. of individuals	181	109
Age (years)	75.1±5.3	73.4±4.8
Body mass index (kg/m^2^)	23.8±3.0	23.1±3.4
Level of education, years (%)		
≤12	53.0	75.3
Marital status (%)		
Married	85.6	75.2
Family income ≥¥5,000,000/year (%)	30.4	21.1
Behavioral factors (%)		
Non-smoker	88.4	99.1
No alcohol intake	29.8	71.6
Total number of multiple diseases (n)	1.6±0.9	1.6±0.9
Accelerometer variables (min/day)		
LLPA	113.4±37.5	143.5±42.3
HLPA	176.5±66.9	241.3±65.9
MPA	50.9±37.6	48.1±27.3
Physical function outcomes		
Timed Up-and-Go test (sec)	6.1±1.2	6.5±1.4
One-leg standing time (sec)	41.8±21.6	44.2±22.1
Maximum gait speed (m/sec)	1.8±0.3	1.7±0.3

Values are presented as mean ± standard deviation or population-weighted percentage. LLPA, low light-intensity physical activity; HLPA, high light-intensity physical activity; MPA, moderate-intensity physical activity.

## Results

### Participants

Participants’ sociodemographic and behavioral factors are presented in Table 1. The 290 participants were divided into two groups according to sex. In the male group, positive correlations were observed for LLPA and HLPA (r = 0.47) and for HLPA and MPA (r = 0.40). However, there was no correlation observed for LLPA and MPA (r = 0.007). In the female group, positive correlations were observed for LLPA and HLPA (r = 0.43) and for HLPA and MPA (r = 0.47), but no correlation was observed for LLPA and MPA (r = -0.001).

### Relationship of intensity of physical activity with physical performance

Following adjustment for potential confounders, MPA was shown to be significantly beneficially associated with the respective physical performance indicators of TUG, OLST, and MGS in the male group (adjusted r-squared values: 0.285, 0.299, and 0.260) and female group (adjusted r-squared values: 0.386, 0.285, and 0.352) (Tables 2 and 3). HLPA was not shown to be significantly beneficially associated with the respective physical performance indicators of TUG, OLST, and MGS in the male group (adjusted r-squared values: 0.165, 0.231, and 0.144) (Table 2) or with OLST in the female group (adjusted r-squared value: 0.202) (Table 3). HLPA showed significant associations with the TUG test and MGS in the female group (adjusted r-squared values; 0.378 and 0.317, respectively) only. In addition, there was no significant association between LLPA and the respective physical performance indicators of TUG, OLST, and MGS in either the male group (adjusted r-squared values: 0.162, 0.227, and 0.147) (Table 2) or the female group (adjusted r-squared values: 0.236, 0.207, and 0.243) (Table 3).

**Table 2:** Association of physical activity and physical performance parameters in males.

Variable	LLPA (SD, 37.5 min)	HLPA (SD, 66.9 min)	MPA (SD, 37.6 min)
Timed Up-and-Go test (sec)	0.011, -0.004~0.005, 0.87*	-0.054, -0.004~0.002, 0.461	**-0.321, -0.015~-0.006, 0.001**
One-leg standing time (sec)	0.085, -0.030~0.129, 0.223	0.104, -0.011~0.078, 0.138	**0.217, 0.042~0.208, 0.003**
Maximum gait speed (m/sec)	-0.067, -0.002~0.001, 0.364	0.030, -0.001~0.001, 0.687	**0.310, 0.001~0.004, 0.001**

Models adjusted for sociodemographic and behavioral covariates were retained through backward elimination.

*Values are presented as standardized β coefficient, non-standardized B 95% confidence interval (CI), and P-value (β, B 95% CI, P).

LLPA, low light-intensity physical activity; HLPA, high light-intensity physical activity; MPA, moderate-intensity physical activity; SD, standard deviation.

**Table 3:** Association of physical activity and physical performance parameters in females.

Variable	LLPA (SD, 42.3 min)	HLPA (SD, 65.9 min)	MPA (SD, 27.3 min)
Timed Up-and-Go test (sec)	-0.059, -0.008~0.004, 0.553*	**-0.399, -0.011~-0.004, 0.001**	**-0.473, -0.031~-0.014, 0.001**
One-leg standing time (sec)	0.038, -0.080~0.119, 0.695	0.082, -0.039~0.093, 0.417	**0.252, 0.048~0.355, 0.011**
Maximum gait speed (m/sec)	0.138, 0.000~0.002, 0.158	**0.272, 0.000~0.002, 0.006**	**0.396, 0.002~0.006, 0.001**

Models adjusted for sociodemographic and behavioral covariates were retained through backward elimination.

*Values are presented as standardized β coefficient, non-standardized B 95% confidence interval (CI), and P-value (β, B 95% CI, P).

LLPA, low light-intensity physical activity; HLPA, high light-intensity physical activity; MPA, moderate-intensity physical activity; SD, standard deviation.

## Discussion

To our knowledge, this is the first time that objectively measured differences in the intensities of physical activity and physical performance have been evaluated according to sex in an elderly Japanese population of men and women. Although it is known that LLPA and HLPA substantially contribute to total energy expenditure, it remains unknown whether there is an association of these intensities of physical activity with physical performance or whether the associations vary with the intensities of physical activity according to sex.

A previous study indicated a beneficial association of LLPA, HLPA, and MPA with biomarkers of cardiometabolic risk such as triglycerides, cholesterol, and insulin in a large population-based study of adults [14]. The overall pattern of these results was consistent with increased intensity being associated with greater cardiometabolic benefits [14]. These findings also reinforce long-standing recommendations for MPA and recent public health messages that participation in MPA may be additionally beneficial to the cardiometabolic health of the overall population [14, 16].

Buman et al. [27] previously suggested that LLPA and HLPA were beneficially related to self-reported physical health and well-being in elderly adults. In the present study, although there were no significant associations detected between LLPA and physical performance in either the male or female participants, increasing LLPA rather than HLPA may be more beneficially associated with self-reported physical health and well-being.

Following adjustment for potential confounders, MPA was observed to be significantly beneficially associated with all physical performance indicators in the male and female participants. Thus, this result may support the notion that MPA not only has cardiometabolic benefits but also improves physical performance in elderly populations. Although the degree of benefit cannot be ascertained in terms of improved physical performance, at least some benefit can be expected from increasing the time spent performing MPA.

In the present study, only the intensity of HLPA showed significant associations with TUG and MGS in the female, but not in the male, participants. The lower physical performance of non-disabled elderly women leaves them at greater risk for subsequent disabling and co-morbid conditions, nursing home admission, and mortality than their male counterparts [28–31]. In comparison with elderly men, elderly women were found to have impaired exercise performance in the domains of lower extremity strength, balance, and ambulation [28–31]. Thus, elderly female populations might more easily experience problems with locomotion compared with their male counterparts.

### Study limitations

Limitations of this study include its small sample size and the single 7-day period over which measurements of physical activity occurred. We did not consider the effect of the time the accelerometer was worn for light-intensity physical activity. Thus, these deficiencies should be addressed in subsequent longitudinal studies.

The study participants were chosen by random sampling. However, the final response rate was very low. Thus, we need to reconsider the reliability and validity of the study participants and/or possible selection bias in a future trial.

Based on the characteristics of the study population, the elderly adults in this study were extremely healthy and active (50.9 ± 37.6 min of MPA per day in the men and 48.1 ± 27.3 min in the women; Table 1). These results may not be generalizable to elderly adults who do less MPA per day or who are less healthy. Nevertheless, the values for physical activity and physical performance that were determined may be important in future research and may also represent appropriate sex-based values for improvements in elderly Japanese populations.

## Conclusions

The present study findings provide novel epidemiological evidence for the potential benefits of HLPA in women. The results also reinforce the potential benefits of MPA in both sexes, which is the mainstay of public health recommendations. Future longitudinal studies are necessary to evaluate the effect of improvements in these values in elderly populations according to sex, and long-term follow-up will be required to evaluate the potential time-related benefits of this improvement.
